# Respiratory syncytial virus vaccination among US adults aged ≥60 years

**DOI:** 10.3389/fimmu.2024.1427550

**Published:** 2024-12-03

**Authors:** Xiaozhen Geng, Wenjun Wang

**Affiliations:** Department of Infectious Diseases, Second Affiliated Hospital of Xi’an Jiaotong University, Xi’an, China

**Keywords:** respiratory syncytial virus, RSV, vaccine, older adults, socioeconomic

## Abstract

**Background:**

Respiratory syncytial virus (RSV) vaccines have been recommended for US adults aged ≥60 years for nearly one year. However, the extent of vaccination coverage and the factors influencing uptake remain underexplored. This study aimed to evaluate national and state-specific RSV vaccination coverage among US adults aged ≥60 years and to identify demographic and socioeconomic factors that influence vaccination uptake.

**Methods:**

This cross-sectional study analyzed data from the US Census Household Pulse Survey, from January 9 to March 4, 2024, which included 49,322 adults aged ≥60 years. Participants self-reported their RSV vaccination status along with demographic and socioeconomic information. Multivariable Poisson regression was used to identify factors associated with vaccination uptake.

**Results:**

As of March 4, 2024, RSV vaccination coverage among US adults aged ≥60 years was estimated at 22.2% (95% CI, 21.6%-22.7%). State-specific vaccination coverage ranged from 12.4% in Mississippi to 33.1% in Colorado. Coverage was higher in older age groups, with 21.2% among those aged 65-69 years, 28.1% among 70-74 years, 31.9% among 75-79 years, and 30.2% among those aged ≥80 years, compared to 10.8% among those aged 60-64 years. Racial/ethnicity disparities were evident, with lower coverage among Hispanics (18.3%) and non-Hispanic Blacks (17.6%) compared to non-Hispanic Whites (23.3%). Coverage was similar between males (22.6%) and females (21.8%). Factors positively associated with vaccination included older age, higher education level, higher household income, unemployment, and residing in the Midwest or West. Conversely, being non-Hispanic Black, uninsured, experiencing financial difficulties, and lack of COVID-19 vaccination were associated with reduced likelihood of receiving the RSV vaccine.

**Conclusions:**

During the first RSV season after the recommendations for RSV vaccination in US adults aged ≥60 years, uptake shows promise but is marked by significant disparities related to race/ethnicity, socioeconomic status, and geographic location. Efforts to address these disparities are crucial to enhance vaccination coverage and reduce the impact of RSV on this vulnerable population.

## 1 Introduction

Respiratory Syncytial Virus (RSV) represents a substantial public health challenge in the United States, particularly among older adults. It manifests across a broad spectrum of acute respiratory illnesses, ranging from mild symptoms to severe lower respiratory tract diseases (1). Annually, RSV is estimated to result in approximately 4.3 million symptomatic cases of acute respiratory illnesses, leading to 1.4 million medical visits, 172,000 hospitalizations, 31,000 intensive care unit admissions, and 15,000 deaths among adults aged ≥60 years (2). Furthermore, the economic burden of RSV is significant (3), with hospitalization costs comparable to those of influenza for patients in this age group (4).

In response to this health threat, three RSV vaccines (Arexvy [GSK], Abrysvo [Pfizer], and mResvia [Moderna]) received approval from the US Food and Drug Administration for the prevention of RSV-associated lower respiratory tract diseases in adults aged ≥60 years (5–7). The Advisory Committee on Immunization Practices (ACIP) recommended a single dose of any FDA-approved RSV vaccine for all adults aged ≥75 years and for adults aged 60-74 years who are at increased risk for severe RSV disease (8). Modeling studies suggested that widespread vaccination of this population could significantly reduce the overall burden of the disease, although the extent of this reduction largely depends on achieving high coverage rates (3, 9).

The US Census Household Pulse Survey (HPS), originally designed to assess the social and economic impact of the COVID-19 pandemic, began including RSV vaccination data in January 2024, offering a valuable source for estimating coverage rates. The primary objective of this study is to use data from the HPS to investigate the recent national and state-specific RSV vaccination coverage among adults aged ≥60 years. By identifying factors independently associated with vaccination uptake, this study aims to uncover effective strategies to enhance vaccine coverage, thereby mitigating the impact of RSV on this vulnerable population.

## 2 Methods

### 2.1 Study sample

The HPS is a nationally representative online survey of US households across all 50 states and the District of Columbia (10). It solicits responses from one adult aged ≥18 years per household, who answers a series of questions over approximately 20 minutes regarding demographic, social, and economic information. The survey incorporates weighting adjustments to address nonresponse bias, household size, and coverage to ensure data representativeness (11, 12). This cross-sectional analysis used data from cycles 1 and 2 of Phase 4.0 of the HPS, conducted from January 9 to March 4, 2024. Response rates for these cycles were 6.5% and 6.8%, respectively. The study sample included 49,322 participants aged ≥60 years who provided RSV vaccination status. Given the public access and de-identified nature of the data, Institutional Review Board approval was not sought. This study adhered to the Strengthening the Reporting of Observational Studies in Epidemiology (STROBE) guideline.

### 2.2 RSV vaccination

In the HPS, participants aged ≥60 years were asked: “There is a vaccine that was recently recommended for some people that helps prevent the respiratory virus called RSV. Have you received the RSV vaccine?” Responses were categorized as “yes” or “no.” Participants who failed to report their vaccination status were excluded from the analysis to avoid misclassification.

### 2.3 Other study measures

Demographic and socioeconomic characteristics collected included age, sex, race/ethnicity (non-Hispanic White, Hispanic, non-Hispanic Black, non-Hispanic Asian, or Non-Hispanic other/multiple races), marital status (married, widowed/separated/divorced, or never married), education level (less than high school, high school, some college or associate degree, or college graduate or above), employment status in the last 7 days (employed or not employed), living region of US (Northeast, South, Midwest, or West), health insurance status (insurance, or no insurance), annual household income (<$25,000; $25,000-$49,999; $50,000-$99,999; or ≥$100,000), difficulty with expenses in the last 7 days (not at all difficult, a little difficult, somewhat difficult, or very difficult), and COVID-19 vaccination status (received or not).

### 2.4 Statistical analysis

The demographic and socioeconomic characteristics of participants were summarized using weighted percentages. National RSV vaccination coverage was estimated for all adults aged ≥60 years and within predefined categories such as age group (60-64 years, 65-69 years, 70-74 years, 75-79 years, and ≥80 years), sex, race/ethnicity, education level, marital status, household income, living region, employment status, health insurance status, difficulty with expenses, and COVID-19 vaccination status. Poisson regression, incorporating all aforementioned variables, was used to generate adjusted prevalence ratios and to identify demographic and socioeconomic characteristics associated with RSV vaccination among adults aged ≥60 years. A complete case analysis approach was used as participants with missing data represented only a small fraction of the total sample. State-specific RSV vaccination coverage was also calculated.

Survey weights were applied to all calculations to ensure nationally representative estimates. Statistical significance was defined as *P <*0.05 (two-tailed). All analyses were performed using StataSE 15 software (StataCorp).

## 3 Results

### 3.1 Participant characteristics

Between January 9 and March 4, 2024, the HPS interviewed a total of 57,634 adults aged ≥60 years. After excluding 8,312 respondents (12.3%) who did not provide their RSV vaccination status, the analysis included 49,322 participants (**Supplementary Figure 1**). Of these, 22,527 (47.0%) were men and 26,795 (53.0%) were women. Participants aged 60-64 years constituted 26.7% of the study population. Regarding racial composition, 73.5% of participants identified as non-Hispanic White, 10.3% as Hispanic, 9.6% as non-Hispanic Black, 3.5% as non-Hispanic Asian, and 3.1% as non-Hispanic of other or multiple races (**Table 1**). No participants were missing data on age, sex, race/ethnicity, education, and geographic region. Minimal data were missing on marital status (0.4%), household income (3.7%), employment (0.7%), financial difficulties (0.1%), health insurance status (1.3%), and COVID-19 vaccination status (0.4%).

**Table 1:** Sample characteristics among US adults aged ≥60 years, Household Pulse Survey, January 09–March 04, 2024.

Characteristic	Sample, (n)	Weighted, %
Total	49,322	
Age (years)		
60-64	12,020	26.7
65-69	13,268	28.6
70-74	11,639	22.7
75-79	7,497	13.4
≥80	4,898	8.6
Sex		
Male	22,527	47.0
Female	26,795	53.0
Race/ethnicity		
Non-Hispanic White	40,154	73.5
Hispanic	2,778	10.3
Non-Hispanic Black	3,441	9.6
Non-Hispanic Asian	1,399	3.5
Non-Hispanic other/multiple races	1,550	3.1
Education		
Less than high school	1,067	7.7
High school	7,365	32.0
Some college or associate degree	16,510	27.7
College graduate or above	24,380	32.5
Marital status		
Married	27,683	62.5
Widowed/divorced/separated	17,726	31.3
Never married	3,731	6.2
Annual household income, $		
<25,000	6,601	16.5
25,000-49,999	11,717	27.8
50,000-99,999	15,230	30.3
≥100,000	13,934	25.5
Geographic region		
Northeast	7,366	17.7
South	16,114	38.0
Midwest	10,214	21.5
West	15,628	22.8
Employment status (last 7 days)		
Employed	15,832	31.4
Not employed/not in work force	33,125	68.6
Health insurance status		
Insurance	47,744	97.3
No insurance	948	2.7
Difficulty with expenses (last 7 days)		
Not at all difficult	27,068	47.1
A little difficult	12,193	27.5
Somewhat difficult	6,591	16.5
Very difficult	3,416	8.9
Received COVID-19 vaccine		
Yes	44,568	88.6
No	4,559	11.4

### 3.2 RSV vaccination coverage

RSV vaccination coverage was estimated at 22.2% (95% CI, 21.6%, 22.7%) among US adults aged ≥60 years, as of March 4, 2024, during the first RSV season after the ACIP recommendations. Vaccination rates varied significantly by age, with coverage of 21.2% in those aged 65-69 years, 28.1% in those aged 70-74 years, 31.9% in those aged 75-79 years, and 30.2% in those aged 80 years and above, compared to 10.8% in those aged 60-64 years. Racial disparities in vaccination rates were also observed, with coverage among Hispanics (18.3%) and non-Hispanic Blacks (17.6%) lower than non-Hispanic Whites (23.3%). In contrast, vaccination rates for non-Hispanic Asians (22.5%) and non-Hispanic others or multiple races (22.2%) were comparable to non-Hispanic Whites (23.3%). The vaccination rates were similar between males (22.6%) and females (21.8%). Higher vaccination rates were noted among those who were unemployed, lived in the Western US, and had higher levels of education and household income. Conversely, lower vaccination rates were associated with financial difficulties, lack of a spouse, unemployment, and lack of insurance. The vaccination rate among those who had not received the COVID-19 vaccine was markedly lower (2.8%) compared to those who had (24.7%) (**Table 2**).

**Table 2:** Respiratory syncytial virus vaccination coverage among adults aged ≥60 years by selected demographic and socioeconomic characteristics, Household Pulse Survey, January 09–March 04, 2024.

Characteristic	RSV vaccination coverage, % (95% CI)	Difference, % (95% CI)
Total	22.2 (21.6, 22.7)	NA
Age (years)		
60-64	10.8 (10.0, 11.5)	ref
65-69	21.2 (20.1, 22.3)	10.4 (9.1, 11.7)^*^
70-74	28.1 (26.8, 29.3)	17.3 (15.8, 18.8)^*^
75-79	31.9 (30.2, 33.6)	21.1 (19.4, 22.9)^*^
≥80	30.2 (28.1, 32.3)	19.4 (17.5, 21.3)^*^
Sex		
Male	22.6 (21.7, 23.4)	ref
Female	21.8 (21.1, 22.5)	−0.7 (−1.9, 0.4)
Race/ethnicity		
Non-Hispanic White	23.3 (22.7, 23.9)	ref
Hispanic	18.3 (15.7, 20.9)	−5.0 (−7.8, −2.2)^*^
Non-Hispanic Black	17.6 (15.8, 19.5)	−5.6 (−7.6, −3.7)^*^
Non-Hispanic Asian	22.5 (19.0, 26.0)	−0.8 (−4.4, 2.8)
Non-Hispanic other/ multiple races	22.2 (18.4, 26.1)	−1.0 (−4.9, 2.8)
Education		
Less than high school	14.6 (11.2, 17.9)	ref
High school	18.5 (17.4, 19.6)	3.9 (0.2, 7.6)^*^
Some college or associate degree	20.3 (19.5, 21.2)	5.7 (2.4, 9.1)^*^
College graduate or above	29.2 (28.5, 29.9)	14.6 (11.2, 18.1)^*^
Marital status		
Married	23.3 (22.6, 24.0)	ref
Widowed/ divorced/separated	20.2 (19.5, 21.0)	−3.1 (−4.1, −2.0)^*^
Never married	20.2 (17.7, 22.7)	−3.1 (−5.5, −0.6)^*^
Annual household income, $		
<25,000	16.0 (14.4, 17.5)	ref
25,000-49,999	19.9 (18.6, 21.1)	3.9 (1.9, 5.9)^*^
50,000-99,999	23.9 (23.0, 24.8)	7.9 (6.1, 9.8)^*^
≥100,000	27.0 (25.8, 28.2)	11.0 (9.1, 13.0)^*^
Geographic region		
Northeast	21.7 (20.3, 23.1)	ref
South	20.0 (19.1, 21.0)	−1.6 (−3.3, 0.1)
Midwest	23.0 (22.0, 24.0)	1.3 (−0.6, 3.2)
West	25.4 (24.1, 26.7)	3.7 (1.7, 5.7)^*^
Employment status (last 7 days)		
Employed	16.3 (15.5, 17.1)	ref
Not employed/not in work force	24.8 (24.1, 25.6)	8.6 (7.4, 9.7)^*^
Health insurance status		
Insurance	22.8 (22.2, 23.3)	ref
No insurance	8.3 (4.9, 11.7)	−14.5 (−18.1, −10.9)^*^
Difficulty with expenses (last 7 days)		
Not at all difficult	27.5 (26.7, 28.4)	ref
A little difficult	18.3 (17.2, 19.3)	−9.2 (−10.6, −7.9)^*^
Somewhat difficult	17.4 (15.9, 18.9)	−10.1 (−11.8, −8.4)^*^
Very difficult	14.8 (12.7, 16.8)	−12.7 (−15.1, −10.4)^*^
Received COVID-19 vaccine		
Yes	24.7 (24.1, 25.2)	ref
No	2.8 (1.9, 3.8)	−21.8 (−23.0, −20.6)^*^

^*^Represents significant difference from the reference.

### 3.3 Factors associated with RSV vaccination

In multivariable analysis, factors independently associated with an increased likelihood of RSV vaccination among adults aged ≥60 years included older age, higher education level (college graduate or above), higher household income (≥$50,000), unemployment, and residence in the Midwest or West regions. Conversely, being non-Hispanic Black, uninsured, financial difficulties, and unvaccinated against COVID-19 were factors associated with a decreased likelihood of RSV vaccination (**Table 3**).

**Table 3:** Factors associated with respiratory syncytial virus vaccination among US adults aged ≥60 years, Household Pulse Survey, January 09–March 04, 2024.

Characteristic	Adjusted prevalence ratio (95% CI)
Age (years)	
60-64	ref
65-69	1.80 (1.64, 1.96)^*^
70-74	2.22 (2.04, 2.42)^*^
75-79	2.37 (2.17, 2.60)^*^
≥80	2.36 (2.14, 2.60)^*^
Sex	
Male	ref
Female	1.00 (0.95, 1.06)
Race/ethnicity	
Non-Hispanic White	ref
Hispanic	0.97 (0.84, 1.12)
Non-Hispanic Black	0.89 (0.80, 0.99)^*^
Non-Hispanic Asian	0.87 (0.75, 1.01)
Non-Hispanic other/ multiple races	1.12 (0.93, 1.35)
Education	
Less than high school	ref
High school	1.14 (0.91, 1.42)
Some college or associate degree	1.15 (0.94, 1.41)
College graduate or above	1.39 (1.12, 1.72)^*^
Marital status	
Married	ref
Widowed/ divorced/separated	0.99 (0.94, 1.05)
Never married	1.08 (0.96, 1.21)
Annual household income, $	
<25,000	ref
25,000-49,999	1.06 (0.95, 1.19)
50,000-99,999	1.17 (1.06, 1.30)^*^
≥100,000	1.28 (1.14, 1.44)^*^
Geographic region	
Northeast	ref
South	0.98 (0.91, 1.07)
Midwest	1.09 (1.00, 1.19)^*^
West	1.15 (1.06, 1.26)^*^
Employment status (last 7 days)	
Employed	ref
Not employed/not in work force	1.26 (1.18, 1.35)^*^
Health insurance status	
Insurance	ref
No insurance	0.64 (0.41, 1.00)^*^
Difficulty with expenses (last 7 days)	
Not at all difficult	ref
A little difficult	0.83 (0.78, 0.89)^*^
Somewhat difficult	0.87 (0.80, 0.96)^*^
Very difficult	0.87 (0.75, 1.00)^*^
Received COVID-19 vaccine	
Yes	ref
No	0.15 (0.10, 0.22)^*^

^*^Represents significant difference from the reference.

### 3.4 State-specific RSV vaccination

**Figure 1:**
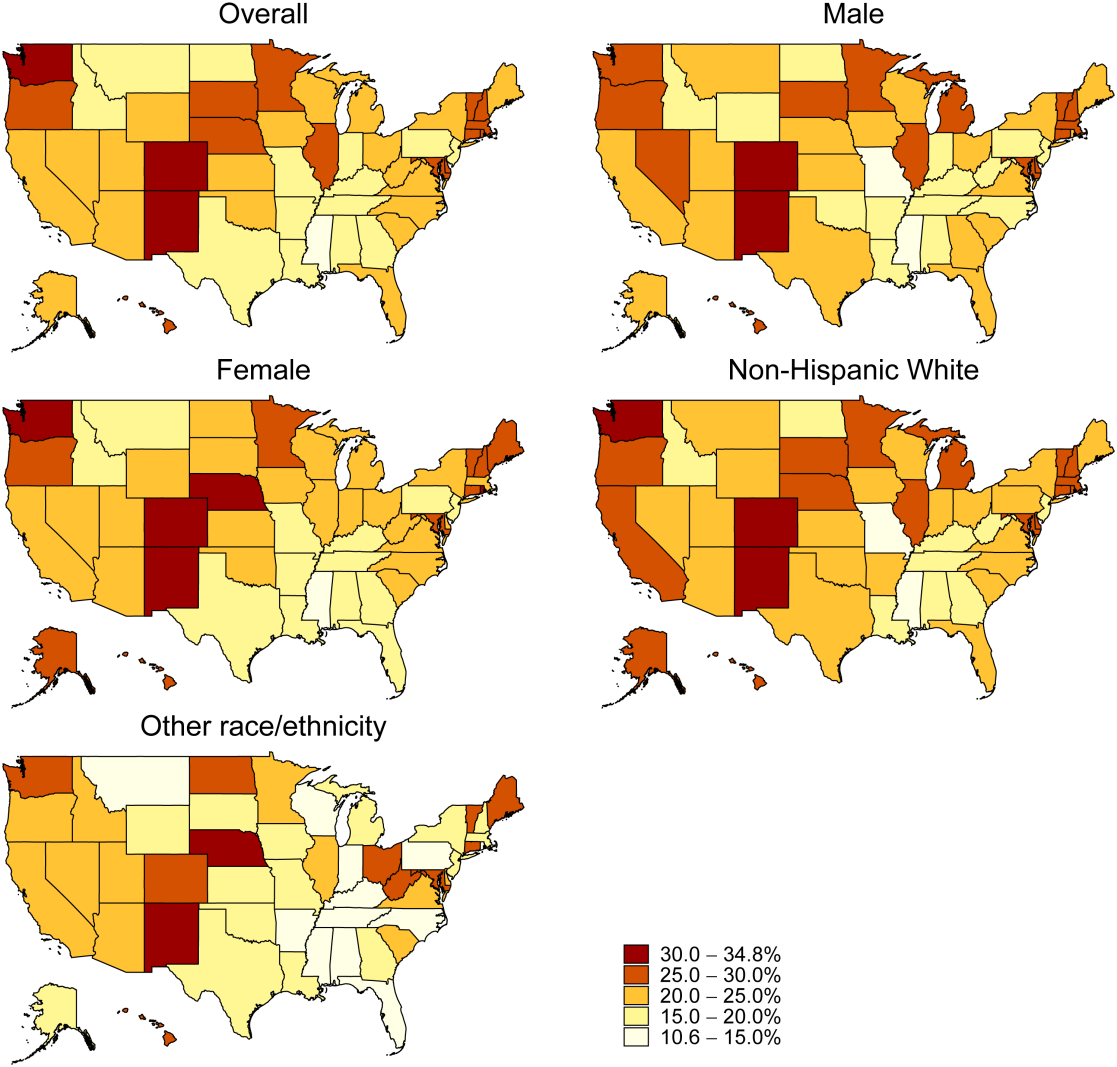
Respiratory Syncytial Virus Vaccination Coverage by State, Household Pulse Survey, January 09–March 04, 2024.

The analysis of state-specific RSV vaccination coverage revealed a wide range of coverage rates across the 50 states and the District of Columbia, from as low as 12.4% in Mississippi to as high as 33.1% in Colorado, with a median coverage rate of 23.1%. Fifteen states (Montana, Texas, Pennsylvania, Indiana, Arkansas, North Dakota, New Jersey, Louisiana, Georgia, Idaho, Tennessee, Kentucky, Alabama, Missouri, and Mississippi) had vaccination rates below 20%. Only three states (Colorado, New Mexico, and Washington) had rates above 30%. RSV vaccination coverage was significantly higher among non-Hispanic Whites compared to other races in thirteen states: Arkansas, California, Colorado, Florida, Illinois, Massachusetts, Michigan, New York, North Carolina, Pennsylvania, Rhode Island, Tennessee, and Wisconsin. No significant differences in vaccination rates between males and females were observed across any state or the District of Columbia (**Figure 1**; **Supplementary Table 1**).

## 4 Discussion

This study, based on the Household Pulse Survey (HPS), found that self-reported RSV vaccination coverage among US adults aged ≥60 years was 22.2% as of March 4, 2024, during the first RSV season after the recommendations. This closely matched the 22.4% estimate from the National Immunization Survey-Adult COVID Module (NIS-ACM) reported one month earlier, in February 2024. The NIS-ACM, designed to monitor self-reported influenza, COVID-19, and RSV vaccination coverage, is also a nationwide representative survey of noninstitutionalized US adults (13). The similarity between these estimates from two independent surveys suggests reliable measurement of RSV vaccination coverage in this age group. What’s more, as both surveys are updated regularly, they offer valuable resources for ongoing RSV vaccination rate monitoring.

The rapid adoption of RSV vaccines, following recommendations by ACIP less than a year earlier (14), contrasts sharply with the historical uptake rates of vaccines such as the pneumococcal and herpes zoster vaccines, which required up to eight and six years, respectively, to achieve comparable coverage levels (15). With over 50% of adults aged ≥60 years expressing intent to receive the RSV vaccine (16), coverage rates are expected to rise further. In 2024, RSV vaccination questionnaire was newly added to the HPS and will be repeated bi-weekly. Thus, this study represents a baseline estimate for RSV coverage and the HPS provides a robust mechanism for tracking vaccination trends.

For over fifty years, the license of an RSV vaccine was hampered by the poor efficacy of early candidates (17). Current development efforts for protein-subunit vaccines have concentrated on the RSV fusion (F) glycoprotein, particularly its metastable prefusion form (preF) (18, 19). Recently, phase 3 trials have demonstrated acceptable safety profiles and satisfactory efficacy of prefusion F protein-based vaccines (20–22), prompting the ACIP to recommend their use for preventing RSV-related acute respiratory illnesses in older adults (8, 14). Initially, ACIP recommended a single dose for adults aged ≥60 based on shared clinical decision-making (14); in June 2024, this was updated to a single dose for all adults aged ≥75 and those aged 60-74 at higher risk for severe RSV disease (8). Despite strong clinical evidence potentially boosting physician recommendations, significant barriers to vaccination remain, particularly the high cost. The RSVPreF3 (Arexvy, GSK) and RSVpreF (Abrysvo, Pfizer) are priced at $280.00 and $295.00 per dose, respectively. These costs significantly exceed those of pneumococcal ($117-$262), herpes zoster ($198), and influenza ($19-$32) vaccines (23). An internet and mail survey highlighted out-of-pocket expenses as a primary barrier for uninsured individuals (24), a finding corroborated by this study’s lower vaccination coverage among uninsured adults (8.3%) compared to insured adults (22.8%).

This study also found pronounced racial and ethnic disparities in RSV vaccination rates, with coverage among non-Hispanic Blacks significantly lower than among non-Hispanic Whites. This race and ethnic disparities in vaccination uptake exist in almost all other vaccines for adult, such as vaccines for COVID-19, influenza, herpes zoster, hepatitis A, hepatitis B, human papillomavirus, and tetanus and diphtheria with acellular pertussis (25, 26). Education attainment, income level, and insurance coverage partly contributed to these disparities. Other factors such as lack of patient awareness, lack of physician recommendation, medical mistrust, and limited English were also noted reasons (26). Some measures are reported to narrow these disparities, such as pharmacist’s active promotion and reminder or recall using telephone or personalized letter (26).

RSV vaccination rates were notably higher among adults aged ≥70 years, with coverage reaching approximately 30%, compared to 10.8% among those aged 60-64 years and 21.2% among those aged 65-69 years. Persons of advanced age represents the most vulnerable demographic when infected with RSV, largely due to compromised immunity and the prevalence of chronic medical conditions such as cardiovascular disease, chronic obstructive pulmonary disease, diabetes, and chronic kidney disease. These factors substantially increase the likelihood of severe respiratory illnesses and hospitalization upon RSV infection (8, 14, 27). Therefore, vaccination against RSV offers substantial benefits to older adults, and prioritizing this population for vaccination is imperative (8, 14).

Low socioeconomic status adversely affects healthcare access, including vaccine uptake among adults (25). This relationship was evident in our study’s univariate and multivariate analyses, which highlighted the impact of education level, household income, health insurance status, and recent financial difficulties on RSV vaccination rates. Data from the National Health Interview Surveys suggest that the implementation of the Affordable Care Act may have reduced socioeconomic disparities in vaccine uptake among US adults aged 18-64 years over the past decade (25). However, this act did not extend Medicaid expansion to adults aged ≥65 years, and thus, socioeconomic disparities in vaccination uptake persist within this older demographic (25). Targeted efforts are necessary to address these disparities among the elderly. Furthermore, our study found a strong correlation between the absence of COVID-19 vaccination and reduced RSV vaccination uptake, indicating shared barriers such as vaccine contraindications, vaccine hesitancy, and issues related to healthcare access.

Variation in RSV vaccine coverage was observed across states, a pattern that is also evident in the uptake of other adult vaccines, such as influenza, pneumococcal, and herpes zoster (28). This variability in vaccine coverage among states likely reflects differences in immunization infrastructure, demographic and socioeconomic factors, as well as state-specific policies (29). These findings underscore the importance of conducting state-specific research to inform targeted interventions that can address these disparities and improve vaccination rates.

Several limitations warrant caution when interpreting these findings. First, the HPS had relatively low response rates (<10%). While weighting adjustments address nonresponse, selection bias remains a potential issue. Second, the reliance on self-reported information may introduce recall and nonresponse biases, affecting data on vaccination uptake, demographics, and socioeconomic factors. Third, the HPS exclusively surveyed noninstitutionalized US adults, thereby excluding populations in institutional settings such as healthcare facilities, prisons, and jails.

In conclusion, this cross-sectional survey, representing the noninstitutionalized adult US population, estimated that RSV vaccination coverage was approximately 22.2% among adults aged ≥60 years as of March 04, 2024, during the first RSV season after the recommendations. The RSV vaccination uptake shows promise but is marked by significant disparities related to race/ethnicity, socioeconomic status, and geographic location. Efforts to address these disparities are crucial to enhance vaccination coverage and reduce the impact of RSV on this vulnerable population.

## Data Availability

The datasets presented in this study can be found in online repositories. The names of the repository/repositories and accession number(s) can be found below: https://www.census.gov/householdpulsedata.
