# Supplementary material for: Respiratory syncytial virus vaccination among US adults aged ≥60 years
Source: Front Immunol. 2024 Dec 3;15:1427550. doi: 10.3389/fimmu.2024.1427550 (PMC11649540; doi:10.3389/fimmu.2024.1427550)
Supplement: Supplementary file 1 [file DataSheet1.docx]

Supplementary Material

# Supplementary Figures


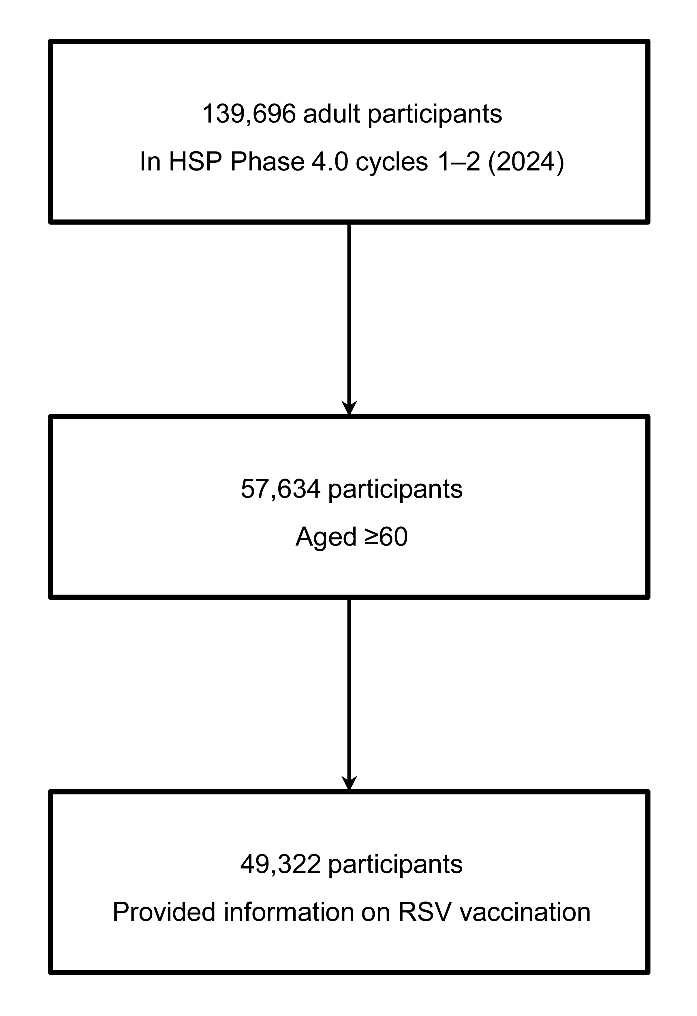


**Supplementary Figure 1.** Determination of the study sample. HSP, the Household Pulse Survey; RSV, respiratory syncytial virus.

# Supplementary Tables

**Supplementary Table 1.** State-Specific Respiratory Syncytial Virus Vaccination Coverage Among Adults Aged ≥60 Years, Household Pulse Survey, January 09–March 04, 2024

| **State** | **Sample size** | **Total, %**  **(95% CI)** | **Sex, % (95% CI)** | | | **Race/ethnicity, % (95% CI)** | | |
| --- | --- | --- | --- | --- | --- | --- | --- | --- |
|  |  |  | **Male** | **Female** | **Difference** | **Non-Hispanic**  **white** | **All other races** | **Difference** |
| Alabama | 752 | 15.9 (12.6, 19.3) | 15.1 (10.1, 20.1) | 16.6 (11.9, 21.3) | 1.5 (-5.5, 8.5) | 16.3 (12.4, 20.1) | 14.8 (8.3, 21.3) | -1.4 (-8.9, 6.1) |
| Alaska | 625 | 24.5 (20.8, 28.2) | 22.0 (16.3, 27.7) | 27.3 (22.0, 32.6) | 5.3 (-3.0, 13.5) | 26.4 (21.7, 31.0) | 18.7 (10.7, 26.8) | -7.7 (-17.6, 2.3) |
| Arizona | 1,626 | 24.2 (21.6, 26.7) | 23.9 (19.8, 28.1) | 24.3 (21.1, 27.6) | 0.4 (-4.9, 5.7) | 24.7 (22.2, 27.1) | 22.5 (15.8, 29.1) | -2.2 (-8.9, 4.5) |
| Arkansas | 626 | 18.9 (15.5, 22.3) | 19.3 (13.9, 24.8) | 18.6 (14.3, 22.9) | -0.8 (-7.8, 6.3) | 20.6 (16.5, 24.7) | 11.8 (6.6, 17.0) | -8.8 (-15.5, -2.1) |
| California | 3,449 | 23.3 (20.9, 25.8) | 24.8 (21.9, 27.7) | 22.1 (18.6, 25.6) | -2.7 (-7.0, 1.5) | 26.1 (23.3, 28.8) | 20.5 (16.6, 24.3) | -5.6 (-10.2, -1.0) |
| Colorado | 1,215 | 33.1 (29.2, 36.9) | 32.9 (27.0, 38.8) | 33.3 (28.9, 37.6) | 0.4 (-6.5, 7.3) | 34.8 (31.3, 38.2) | 25.5 (16.4, 34.5) | -9.3 (-17.5, -1.0) |
| Connecticut | 808 | 26.0 (21.5, 30.6) | 26.5 (19.2, 33.9) | 25.5 (20.3, 30.8) | -1.0 (-9.8, 7.9) | 25.2 (20.1, 30.4) | 29.1 (17.8, 40.4) | 3.9 (-8.8, 16.6) |
| Delaware | 627 | 26.6 (22.5, 30.7) | 29.4 (23.1, 35.7) | 24.3 (19.0, 29.5) | -5.2 (-13.4, 3.1) | 28.6 (23.7, 33.5) | 21.0 (13.0, 29.0) | -7.7 (-17.3, 1.9) |
| District of Columbia | 254 | 27.9 (20.2, 35.5) | 31.8 (19.8, 43.7) | 24.1 (14.1, 34.0) | -7.7 (-23.8, 8.3) | 37.4 (23.9, 50.9) | 21.8 (11.5, 32.0) | -15.6 (-33.0, 1.8) |
| Florida | 2,153 | 20.1 (17.3, 23.0) | 20.5 (16.6, 24.3) | 19.8 (16.0, 23.6) | -0.7 (-5.8, 4.4) | 22.7 (20.0, 25.3) | 14.7 (8.2, 21.1) | -8.0 (-14.8, -1.2) |
| Georgia | 1,248 | 18.0 (15.1, 20.9) | 20.1 (15.2, 25.0) | 16.2 (13.2, 19.2) | -3.9 (-9.4, 1.6) | 17.9 (15.0, 20.8) | 18.2 (12.5, 23.9) | 0.3 (-5.9, 6.6) |
| Hawaii | 668 | 28.9 (23.5, 34.3) | 27.7 (20.7, 34.7) | 30.0 (22.0, 37.9) | 2.2 (-8.2, 12.7) | 28.8 (18.8, 38.8) | 28.9 (23.4, 34.5) | 0.1 (-10.2, 10.4) |
| Idaho | 882 | 18.0 (14.8, 21.1) | 18.1 (14.3, 22.0) | 17.8 (13.8, 21.8) | -0.4 (-5.0, 4.2) | 17.6 (14.1, 21.1) | 21.9 (10.0, 33.8) | 4.3 (-8.5, 17.2) |
| Illinois | 975 | 25.6 (22.3, 28.9) | 26.9 (22.3, 31.6) | 24.4 (19.0, 29.8) | -2.5 (-10.1, 5.1) | 27.3 (23.4, 31.2) | 20.6 (15.1, 26.2) | -6.7 (-13.3, 0.0) |
| Indiana | 933 | 19.3 (16.1, 22.6) | 17.7 (12.7, 22.6) | 20.8 (16.5, 25.2) | 3.2 (-3.5, 9.8) | 20.5 (17.0, 24.0) | 12.2 (3.9, 20.5) | -8.3 (-17.5, 0.8) |
| Iowa | 796 | 23.6 (20.1, 27.1) | 23.0 (17.3, 28.7) | 24.2 (19.6, 28.8) | 1.2 (-6.4, 8.8) | 23.9 (20.2, 27.7) | 19.0 (4.7, 33.3) | -4.9 (-20.2, 10.4) |
| Kansas | 834 | 23.9 (19.9, 28.0) | 24.8 (18.7, 30.8) | 23.1 (17.8, 28.4) | -1.6 (-9.6, 6.3) | 24.6 (20.3, 29.0) | 19.5 (7.9, 31.0) | -5.2 (-17.5, 7.2) |
| Kentucky | 742 | 17.3 (13.7, 20.9) | 17.1 (11.6, 22.6) | 17.6 (13.6, 21.6) | 0.5 (-5.7, 6.8) | 18.0 (14.0, 21.9) | 12.4 (5.0, 19.9) | -5.5 (-14.1, 3.0) |
| Louisiana | 656 | 18.0 (14.7, 21.4) | 18.0 (13.0, 22.9) | 18.1 (13.2, 22.9) | 0.1 (-7.0, 7.2) | 18.4 (14.6, 22.2) | 17.1 (9.7, 24.5) | -1.3 (-9.7, 7.2) |
| Maine | 628 | 24.4 (20.4, 28.4) | 22.5 (16.0, 29.0) | 26.2 (21.8, 30.7) | 3.8 (-3.8, 11.3) | 24.4 (20.1, 28.6) | 26.3 (9.5, 43.0) | 1.9 (-15.8, 19.6) |
| Maryland | 1,053 | 27.0 (23.6, 30.5) | 29.1 (24.7, 33.5) | 25.2 (20.9, 29.5) | -3.9 (-9.3, 1.4) | 27.5 (23.8, 31.1) | 26.3 (20.5, 32.2) | -1.2 (-7.5, 5.1) |
| Massachusetts | 1,168 | 25.2 (22.1, 28.4) | 27.9 (23.3, 32.4) | 22.9 (18.3, 27.4) | -5.0 (-11.6, 1.6) | 26.8 (23.6, 30.1) | 16.7 (9.1, 24.2) | -10.1 (-18.0, -2.3) |
| Michigan | 1,378 | 24.4 (21.3, 27.6) | 25.8 (20.6, 31.1) | 23.1 (19.4, 26.9) | -2.7 (-9.3, 3.9) | 25.6 (22.2, 29.0) | 18.1 (12.5, 23.8) | -7.4 (-13.7, -1.2) |
| Minnesota | 980 | 25.6 (22.1, 29.0) | 25.7 (21.1, 30.3) | 25.5 (21.5, 29.5) | -0.2 (-5.3, 4.8) | 25.7 (22.0, 29.3) | 24.7 (12.4, 37.0) | -0.9 (-14.0, 12.1) |
| Mississippi | 522 | 12.4 (9.0, 15.9) | 13.3 (7.4, 19.2) | 11.7 (07.8, 15.6) | -1.6 (-8.7, 5.5) | 12.1 (8.2, 15.9) | 13.1 (6.3, 19.9) | 1.0 (-6.9, 8.9) |
| Missouri | 918 | 15.1 (12.5, 17.8) | 14.9 (10.7, 19.1) | 15.3 (11.9, 18.6) | 0.3 (-5.0, 5.6) | 14.6 (12.4, 16.8) | 17.8 (4.8, 30.8) | 3.2 (-10.1, 16.5) |
| Montana | 577 | 19.7 (16.0, 23.4) | 21.4 (15.2, 27.5) | 17.9 (13.0, 22.9) | -3.4 (-11.9, 5.1) | 20.4 (16.7, 24.1) | 10.6 (-1.9, 23.2) | -9.7 (-22.3, 2.8) |
| Nebraska | 639 | 27.3 (22.5, 32.1) | 23.4 (15.6, 31.3) | 31.0 (25.7, 36.2) | 7.5 (-1.6, 16.6) | 26.7 (22.1, 31.3) | 31.1 (8.0, 54.2) | 4.3 (-19.6, 28.3) |
| Nevada | 780 | 23.0 (18.6, 27.3) | 25.1 (18.1, 32.1) | 20.9 (15.4, 26.3) | -4.2 (-13.4, 4.9) | 22.5 (17.5, 27.5) | 24.0 (14.8, 33.2) | 1.5 (-9.3, 12.3) |
| New Hampshire | 809 | 26.5 (21.9, 31.1) | 25.7 (20.0, 31.4) | 27.2 (20.0, 34.3) | 1.5 (-7.7, 10.7) | 26.9 (21.9, 31.9) | 18.2 (0.2, 36.3) | -8.7 (-28.4, 11.0) |
| New Jersey | 828 | 18.8 (15.1, 22.6) | 19.6 (13.5, 25.7) | 18.2 (13.7, 22.6) | -1.4 (-8.8, 5.9) | 18.8 (15.2, 22.5) | 18.7 (10.9, 26.6) | -0.1 (-8.2, 8.0) |
| New Mexico | 1,087 | 33.0 (29.1, 36.9) | 31.6 (25.8, 37.4) | 34.2 (28.9, 39.5) | 2.6 (-5.3, 10.5) | 34.6 (30.3, 38.8) | 31.2 (25.1, 37.3) | -3.4 (-10.4, 3.6) |
| New York | 773 | 21.1 (17.9, 24.2) | 22.2 (16.9, 27.6) | 20.0 (16.4, 23.7) | -2.2 (-8.8, 4.3) | 23.5 (19.7, 27.3) | 16.0 (10.1, 21.9) | -7.5 (-14.8, -0.2) |
| North Carolina | 991 | 20.9 (17.6, 24.2) | 19.8 (14.5, 25.1) | 21.9 (17.2, 26.7) | 2.1 (-5.6, 9.8) | 22.9 (19.1, 26.7) | 14.9 (8.5, 21.3) | -8.0 (-15.3, -0.7) |
| North Dakota | 302 | 18.9 (14.0, 23.7) | 15.1 (9.5, 20.8) | 22.7 (15.3, 30.1) | 7.6 (-1.4, 16.5) | 18.3 (13.2, 23.3) | 27.8 (7.6, 48.0) | 9.5 (-11.4, 30.5) |
| Ohio | 865 | 22.9 (19.3, 26.5) | 21.8 (16.8, 26.8) | 23.7 (18.7, 28.7) | 1.9 (-5.1, 8.9) | 22.4 (18.6, 26.1) | 25.5 (16.4, 34.7) | 3.2 (-6.4, 12.8) |
| Oklahoma | 847 | 20.9 (17.3, 24.5) | 17.2 (12.4, 22.0) | 24.0 (18.7, 29.2) | 6.8 (-0.4, 13.9) | 22.0 (17.1, 27.0) | 16.8 (10.3, 23.3) | -5.2 (-14.7, 4.2) |
| Oregon | 1,323 | 28.6 (25.4, 31.9) | 29.1 (24.0, 34.2) | 28.3 (23.9, 32.6) | -0.8 (-7.8, 6.1) | 29.3 (25.7, 32.9) | 24.1 (17.3, 30.9) | -5.2 (-12.9, 2.5) |
| Pennsylvania | 1,281 | 19.6 (16.5, 22.7) | 19.7 (16.1, 23.2) | 19.6 (15.6, 23.6) | -0.1 (-4.3, 4.2) | 21.2 (17.7, 24.7) | 11.2 (6.7, 15.7) | -10.0 (-15.4, -4.6) |
| Rhode Island | 525 | 26.3 (21.0, 31.5) | 20.4 (14.1, 26.8) | 31.5 (23.0, 40.0) | 11.1 (-0.3, 22.4) | 29.0 (23.5, 34.5) | 10.9 (2.6, 19.2) | -18.1 (-27.8, -8.5) |
| South Carolina | 852 | 20.8 (17.0, 24.6) | 21.6 (15.4, 27.8) | 20.2 (15.5, 24.8) | -1.4 (-9.1, 6.4) | 20.2 (16.0, 24.3) | 22.7 (12.9, 32.4) | 2.5 (-8.4, 13.4) |
| South Dakota | 569 | 25.8 (21.0, 30.7) | 27.2 (19.8, 34.7) | 24.5 (18.0, 31.0) | -2.7 (-12.7, 7.3) | 26.7 (21.6, 31.8) | 18.7 (0.0, 37.4) | -8.0 (-27.6, 11.6) |
| Tennessee | 957 | 17.9 (15.2, 20.5) | 15.5 (11.7, 19.3) | 20.0 (16.4, 23.5) | 4.5 (-0.7, 9.6) | 18.9 (15.9, 22.0) | 13.1 (8.5, 17.7) | -5.8 (-11.2, -0.5) |
| Texas | 2,219 | 19.6 (17.1, 22.2) | 21.2 (17.2, 25.2) | 18.4 (15.6, 21.2) | -2.8 (-7.4, 1.8) | 20.8 (17.5, 24.1) | 17.8 (14.2, 21.5) | -3.0 (-7.7, 1.7) |
| Utah | 1,002 | 21.8 (18.6, 25.0) | 21.1 (16.4, 25.8) | 22.4 (17.3, 27.6) | 1.3 (-6.2, 8.8) | 21.5 (18.0, 24.9) | 24.0 (12.9, 35.1) | 2.5 (-9.3, 14.4) |
| Vermont | 546 | 27.3 (21.9, 32.7) | 28.1 (19.6, 36.6) | 26.5 (20.3, 32.7) | -1.6 (-11.7, 8.5) | 27.2 (21.7, 32.6) | 29.0 (1.2, 56.9) | 1.8 (-26.2, 29.9) |
| Virginia | 1,147 | 23.9 (21.1, 26.6) | 24.7 (20.2, 29.2) | 23.2 (19.1, 27.2) | -1.5 (-8.1, 5.1) | 24.4 (21.0, 27.8) | 22.6 (15.0, 30.1) | -1.8 (-11.0, 7.4) |
| Washington | 1,920 | 30.6 (27.8, 33.5) | 29.9 (26.9, 33.0) | 31.3 (27.3, 35.2) | 1.3 (-2.8, 5.4) | 31.3 (28.9, 33.8) | 27.4 (18.8, 36.0) | -3.9 (-12.2, 4.4) |
| West Virginia | 468 | 20.4 (15.5, 25.3) | 19.2 (11.3, 27.0) | 21.6 (15.0, 28.1) | 2.4 (-8.1, 12.8) | 19.8 (15.3, 24.3) | 26.7 (6.6, 46.8) | 6.9 (-12.6, 26.3) |
| Wisconsin | 1,025 | 23.1 (20.3, 26.0) | 22.3 (17.8, 26.7) | 24.0 (20.1, 27.8) | 1.7 (-4.2, 7.7) | 24.0 (20.9, 27.1) | 14.4 (5.5, 23.2) | -9.7 (-19.2, -0.1) |
| Wyoming | 474 | 20.9 (15.9, 25.9) | 17.7 (12.0, 23.5) | 24.0 (16.4, 31.5) | 6.2 (-2.9, 15.3) | 21.2 (15.8, 26.7) | 15.4 (-6.2, 36.9) | -5.9 (-29.1, 17.3) |
